# Chat agents respond more empathetically by using hearsay experience

**DOI:** 10.3389/frobt.2023.960087

**Published:** 2023-07-25

**Authors:** Hiromi Narimatsu, Hiroaki Sugiyama, Masahiro Mizukami, Tsunehiro Arimoto

**Affiliations:** NTT Communication Science Laboratories, Nippon Telegraph and Telephone, Kyoto, Japan

**Keywords:** dialogue system, empathy, conversational strategy, chatbot, user experience understanding, having similar experience, convey empathy, conversational agent

## Abstract

As the responses of chat dialogue systems have become more natural, the empathy skill of dialogue systems has become an important new issue. In text-based chat dialogue systems, the definition of empathy is not precise, and how to design the kind of utterance that improves the user’s impression of receiving empathy is not clear since the main method used is to imitate utterances and dialogues that humans consider empathetic. In this study, we focus on the necessity of grasping an agent as an experienceable Other, which is considered the most important factor when empathy is performed by an agent, and propose an utterance design that directly conveys the fact that the agent can experience and feel empathy through text. Our system has an experience database including the system’s pseudo-experience and feelings to show empathetic feelings. Then, the system understands the user’s experiences and empathizes with the user on the basis of the system’s experience database, in line with the dialogue content. As a result of developing and evaluating several systems with different ways of conveying the aforementioned rationale, we found that conveying the rationale as a hearsay experience improved the user’s impression of receiving empathy more than conveying it as the system’s own experience. Moreover, an exhaustive evaluation shows that our empathetic utterance design using hearsay experience is effective to improve the user’s impression about the system’s cognitive empathy.

## 1 Introduction

As the performance of natural language processing technology improves (Vaswani et al., 2017; Radford et al., 2019; Raffel et al., 2019), the responses of text-based dialogue systems to open-domain conversations, such as daily conversations, are becoming closer to human responses (Li et al., 2016; Kottur et al., 2017; Adiwardana et al., 2020; Roller et al., 2020). For example, Google’s Meena and Facebook’s BlenderBot have significantly improved on previous deep learning-based methods by collecting large amounts of datasets of human–human conversations and using them to train large-scale neural-based generative models (Adiwardana et al., 2020; Roller et al., 2020). The results of these models are reported to be as responsive as or better than actual human–human dialogues in terms of context-sensitive utterance generation. Although the performance of neural-based dialogue systems has been improving at a dizzying pace recently, traditional rule-based methods have also been able to produce human-like responses by allowing the system to control thetopic to some extent (Weizenbaum, 1966; DeVault et al., 2014; Augello et al., 2017). For example, a recent competition to evaluate the performance of dialogue systems with about 15 turns of conversation showed that the responses of rule-based dialogue systems were more human-like and natural, even without any restrictions on the user’s response (Higashinaka et al., 2021). In addition, most dialogue systems currently in commercial use, such as conversational agents in smartphones or smart speakers, are rule-based because the response is completely controllable (Nuruzzaman and Hussain, 2018), while the neural-based methods may output responses that are not desired depending on the context, and it is difficult to completely control the output (Adiwardana et al., 2020; Bang et al., 2021).

On the other hand, as the response performance of both rule-based and neural-based systems has become more human-like, it has been pointed out that the skill of *empathy*, which improves the user’s impression of *the system understand/empathize me*, is lacking as an important element of human-likeness in conversation (Shum et al., 2018; Quick, 2021). Two reasons are given by Quick (2021). One is that conventional dialogue systems are evaluated without strictly defining empathy, and therefore, no design guidelines have been provided. Actually, although a study has aimed to make the system’s responses more empathetic (Rashkin et al., 2018), they focused on imitating human–human conversation named *empathetic dialogue dataset* collected by instructing humans to have empathetic conversations with each other and do not provide guidelines for empathetic utterance design. The other is that dialogue systems are often only assessed as cognitive or affective empathy despite the necessity of both cognitive and affective empathy in dialogue systems. Cognitive empathy is to understand the feelings of the other person, and affective empathy is to share the same feelings with the other person; therefore, a user’s impression of the system should be evaluated separately from two perspectives. They also stated the necessity of making users *grasp the robot or agent as an Other who can experience the entity*, because it is a robot/agent (not a human), as a particularly important element.

For making a user grasp robot/agent as *others* who can experience them, there have been studies by using non-verbal behaviors such as facial expressions and gestures to make them appear as if they have emotions (Kühnlenz et al., 2013; McColl and Nejat, 2014). However, in the case of text, this is not easy because it is necessary to express the appropriate emotion at the time according to various contents, which requires comprehensive skills in language comprehension and expression (Ma et al., 2020).

The purpose of this study is to examine the kinds of utterances in text-based conversations that can enhance users’ impressions of receiving empathy from a view point of cognitive and affective empathy in a way that can be incorporated into rule-based dialogue systems. Specifically, we focus on grasping the agent as an experienceable Other, as described previously (Quick, 2021), to design an utterance that directly evokes this feeling and to confirm its effectiveness. One of the straightforward approaches to make the user grasp the agent as an experienceable Other is to show the rationale of “understanding” the user’s experience and feelings as the system’s own experience similar to the user’s experience, such as “I have had a similar experience before” or “I felt enjoyment at that time,” or as a simulated experience within the range that the system itself understands. To realize this approach, we have worked on the design of empathetic utterance generation based on the understood results of the user’s experience and feelings, the system’s experience (as pseudo-experience), and the system’s experience. In this paper, we propose the design of a system utterance that shows the attitude of understanding the user’s experience and empathizing with it based on a common database containing the system’s experience and examine what kind of content (the system’s own experience, hearsay experience of the system, feelings of the system, etc.) included in the utterance is effective to improve the impression of empathy.

**FIGURE 1:**
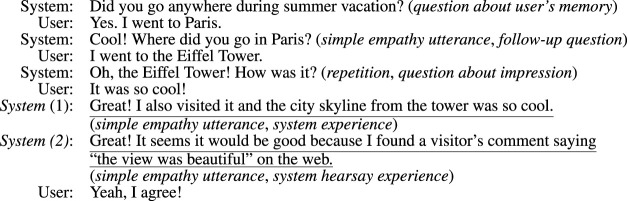
Example dialogue flow of the proposed chat system. System 1 is an example empathetic utterance using the system’s own experience, and System 2 is that using the system’s hearsay experience. The statements in parentheses indicate strategies for the system language. The system repeatedly asks questions about the blank slot in the user’s 5W1H frame and, at the end of the dialogue, asks for the user’s impressions and empathizes with the user based on the system’s experience. The underlined system utterance is an empathetic utterance based on the system’s experience, which is similar to the user’s experience.

The contributions of this paper are as follows:

• A conversational system design is proposed to generate utterances that represent the system’s experiences, which is similar to the user’s experience as a rationale for empathy.
• The effect of including hearsay experiences (example is shown in Figure 1) as a rationale for empathetic utterances has been verified. This improves the user impression of both cognitive and affective empathy, which are written as “understanding” and “empathizing” in the evaluation section, regardless of whether the experiences are similar.
• The effect of including the system’s own feelings/impression in the utterance, as well as the reason for the impression given, such as the circumstances that led to those feelings, is evident in the case where the system’s own experiences are conveyed as a rationale for empathy. This enhances the impression of empathy.

## 2 Related work

For *making the user grasp the system as an experienceable Other*, research has been conducted mainly in the field of human–robot interaction. To show that a system has emotions, recognizing the user’s facial expressions and having the system make similar facial expressions or behave as if it has emotions are typical examples of such research studies (Kühnlenz et al., 2013; McColl and Nejat, 2014). They mainly use nonverbal information such as facial expressions and behavior to make the system appear “emotional,” and the kinds of behavior that make the system appear human have been studied. However, there have not been many studies that assess the language of the system, such as the content of the system’s utterance.

In terms of text and contents of conversation, it is said that in human–human conversation analysis studies, showing that conversationalists have similar experiences has the effect of increasing the impression of receiving empathy (Batson et al., 1996; Eklund and Hansen, 2009). However, “a human being recognized as an experienceable other” and a robot cannot be treated under the same conditions, and it is unclear whether it is necessary to have “had the same experience.” It is also unclear whether it is necessary for the system to have “experienced the same thing.”

Moreover, since there is a high barrier (understanding the user’s experience and having experience in the system) to doing so in language, in practice, the most common method is to imitate conversation between people. For example, to produce empathetic utterances, a method has been proposed that uses utterances annotated with empathy labels, prepared by annotating conversations between people (Higashinaka et al., 2008; Kawahara, 2018). Another method trains deep learning-based generative models using human–human conversation data, and it is conducted by instructing conversational partners to empathize with each other (Rashkin et al., 2018). Although such methods are useful because they can generate utterances that people perceive as empathetic, in actual use, the definition of empathetic utterance is not strict, and it is not clear what kind of utterance improves the impression of empathy.

In the context of mental health, the kinds of utterances that have the effect of empathy have been investigated, but this is different from empathy in daily conversations. For example, in counseling, it is not always a good thing to have empathy or to have had the same experience, and empathy can have the opposite effect (DeVault et al., 2014). In addition, the design of these utterances is basically related to the system’s response when the user is in a negative situation, and the design guidelines are different from those of empathetic utterances, which are effective for various positive and negative events that occur in everyday conversation. For everyday conversation, there is a study that analyzed human–human empathetic dialogues (Rashkin et al., 2018) by annotating utterances in dialogue (Welivita and Pu, 2020). Although they classified the types of utterances that are common into different categories such as questions and acknowledgements, they do not focus on how to construct utterances that directly show empathy.

From these facts, it is not clear which utterances are effective in improving the impression of empathy in daily conversation. In this study, we design a dialogue system that empathizes based on the rationale of similar experiences of the system’s own or hearsay and examine how much the utterances contribute to improving the impression of empathy. We believe that it would be effective to provide a design guideline for what kinds of utterances are effective so that those who are constructing new dialogue systems can generate utterances based on the guideline.

## 3 Empathetic dialogue system

To develop a system that is recognized as an Other who understands the user’s experiences and feelings, we propose an utterance design and its construction method that shows empathy based on the system’s experiences and feelings. We adopt controlling the dialogue flow based on *slot filling* (Bobrow et al., 1977; Weld et al., 2021) to make the system respond to some extent according to the context of the conversation with the user. Since the human–human empathic dialogue dataset contains many questions for the user (Rashkin et al., 2018; Welivita and Pu, 2020), a dialogue system based on asking questions to fill the slot is a reasonable strategy as a design for empathetic dialogue. Then, we apply a 5W1H (who, what, where, when, why, and how) frame (Han et al., 2013) with some modification as a slot to understand the user’s experiences and feelings. We propose to add *impression* to the 5W1H frame (call 5W1H+I given as follows) since the object of empathy is the user’s impression, not *how*. In addition, we decided to add *impression reason* to the 5W1H+1 frame (call 5W1H+II given as follows) to show support for the impression. Using this framework, we can understand the user’s experience and feelings by extracting the part of the user’s expression corresponding to each slot and filling in the slots. We also propose to create the system’s experience database in the same frame as the user’s experience. This makes it easier for the system to find system experiences that are similar to the user’s experience and empathize with the user’s experience. Finally, we adopt a template-based utterance generation approach by filling elements into a predefined utterance template in order to easily generate utterances based on the system’s experience.

**FIGURE 2:**
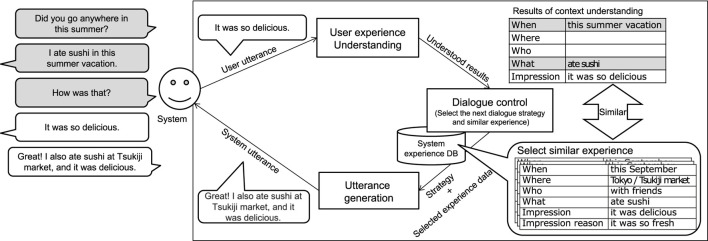
System’s overall operation flow. The square boxes represent modules (user experience understanding module/dialogue control module and utterance generation module). The system comprehends the user’s experience through conversation and understands the results of the item frames as the user’s experience, as shown in the top right table in the user experience understanding module. It then searches its pseudo-experience database and selects an experience that resembles the user’s experience in the dialogue control module. At that time, it sets the dialogue strategy, such as asking questions or conveying empathy, depending on the dialogue context. The utterance templates are related to the dialogue strategy, and an utterance is generated using the template and the selected experience in the utterance generation module.

Figure 2 shows the system’s overall operation flow. In this section, we first describe the design of the dialogue system (Section 3.1) and then explain in detail how to construct the system experience database (Section 3.2) and the methods for building the three modules; user experience understanding module (Section 3.3), dialogue control module (Section 3.4), and utterance generation module (Section 3.5).

### 3.1 System design

Here, we introduce the strategy of the dialogue flow and approaches of empathetic utterance generation based on the system’s experience database. Figure 1 shows an example of the dialogue flow of our proposed system. The basic strategy of conversation follows scenario-based dialogue systems that are generally used in task-oriented dialogue systems (Zhang et al., 2020), but differs from traditional scenario-based systems, in that it requires a correct understanding of the user’s utterances in order to apply scenario-based dialogue to everyday conversations. Therefore, although the system basically takes the initiative by always asking a question about 5W1H+I frames before giving the user the right to speak, it can be described as a hybrid initiative-type system since the content of the system response changes depending on the user’s response.

The system proceeds the conversation to fill in the 5W1H+I frame which is used for understanding the user’s experience. To elicit user experiences, we chose a strategy that conversationally elicits user experiences based on 5W1H frames (Han et al., 2013). Specifically, the system repeatedly asks questions about the blank slot in the user’s 5W1H+I frame and, when all slots except the *impression* slot are filled, asks questions about impressions and then empathizes with the user based on the system’s experience. The overall conversation flow including other candidates’ utterances is as follows: question about user’s memory.

(Did you go anywhere during summer vacation?/Where is the best place you have ever traveled?) → simple empathy utterance (Good!/Cool!), follow-up question based on system’s experience (What did you do there?/Eiffel Tower is famous in Paris. Where did you go in Paris?) → repetition (Oh, the Eiffel Tower!), and question about impression (How was that?) → empathy utterance based on its experience as reason (Yeah, I also visited there and the city skyline from the tower was so cool).

For constructing system’s utterances, we consider using utterance templates with the understood elements of user’s 5W1H+I frames and system’s experience data. For example, when the system wants to ask what the user did at the place they went, it can use the template “What did you do in [(user’s) where]?” to generate “What did you do in Paris?” if [(user’s) where] is recognized as *Paris*. The same applies to the use of the system’s experience database; we simply make the utterance template “I [what] [where]” if the utterance expected to be generated is “I ate sushi in Tokyo.” By this setting, we can simply define the form of the 5W1H+II elements of the system experience database from the template, i.e., *ate sushi* in the *what* frame and *in Tokyo* in the *where* frame.

For using the system’s experience as evidence of empathy, we consider two approaches. One expresses it as the system’s own experience, e.g., “I went to the Eiffel Tower and the view is so cool.” The other expresses hearsay experience, e.g., “Good! I found a visitor’s comment saying, ‘the view is so cool’ on the web.” Although both descriptions are the same from the standpoint of empathy based on the experience of the system, they are expressed differently, i.e., “I went to [where] and [impression]” and “Good! I found a visitor’s comment saying, ‘[impression]’ on the web,” In this way, the template-based utterance generation approach we adopted is suitable for flexibly changing the expression of both approaches using an identical system experience dataset.

In order to take the aforementioned strategy, it is necessary to understand the user’s experiences as 5W1H+I slots through conversation. To understand the elements of 5W1H+I frames from user’s utterances, we develop 5W1H+I phrase recognizers. This study also contributes to understanding user experiences through conversation in chatting situations. The details are described in Section 3.3.

### 3.2 Construction of a system experience database

We use 5W1H+II as the system’s experience data frames. To make workers who create databases easily understand what to write in each frame, we prepared utterance templates and some complete utterance examples filled with elements of the system’s experience database. For example, when the utterance template “I [what] [where]” and completed utterance “I ate sushi in Tokyo” were given, we can easily understand that *ate sushi* is placed in the *what* frame and *in Tokyo* is placed in the *where* frame. Similarly, when the utterance template and completed utterances; “I [what] [who] [impression]” and “I climbed a tower with my family. The view was so cool” were given, we can understand that *climbed a tower* is placed in the *what* frame, *with my family* is placed in the *who* frame, and *The view was so cool* is placed in the *impression* frame. To describe the *impression*’s reason, we prepared the utterance template “[impression] because [impression reason]” and completed utterance “It was delicious because it was so fresh.”

It is important to note that we created 2,652 pseudo-experience data in the *travel* domain in this experiment, but the amount of sufficient experience data will depend on the domain. Since this system experience data are also used as training data for developing a 5W1H+I phrase recognizer in combination with templates, it might be better to have more than one thousand templates depending on the number of templates.

### 3.3 Construction of a user experience understanding module

**FIGURE 3:**
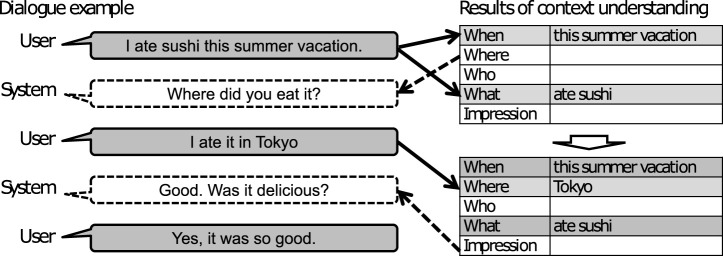
Dialogue example based on results of context understanding.

Although we use a strategy of asking questions based on the framework of the 5W1H+I information as shown in Figure 3, we must understand which word or phrase corresponds to which 5W1H+I. We show three examples in which understanding the user’s utterance is easy or difficult when the system’s question is “Where did you go?” An easy example is the user simply answering the question with “I went to the Eiffel Tower.” A difficult example is the user instead replying to the question with more details, such as “I went to the Eiffel Tower and enjoyed the view from the top.” In this case, the system needs to understand *what* and *impression*. Furthermore, there are some cases in which the user ignores the question and speaks something else such as mentioning, “I ate delicious steak.”

To understand 5W1H+I, including ambiguous expressions whose recognition target is not Named Entity (NE), such as described previously, we developed a 5W1H+I recognizer. Among the 5W1H information, the time and place types of information have been the recognition target of the conventional NE (Nadeau and Sekine, 2007). For example, proper nouns such as *yesterday* and *Tokyo* in “I went to Tokyo yesterday” are extracted as the entities of time and location. However, the information extracted as named entities is insufficient for understanding casual conversations. Previous studies have extracted phrases that are understood as time and location in real human conversations, and they have shown that such phrases (other than proper nouns) accounted for the majority of location phrases (Narimatsu et al., 2018).

Therefore, we developed a phrase recognizer to extract phrases corresponding to 5W1H+I contained in user utterances. We developed this using sequence-labeling methods that are effective for NE recognition. We constructed the model using the sequence-labeling methods of Bidirectional Encoder Representations from Transformers (BERT) (Devlin et al., 2018)^1^, which can be applied to various tasks by fine-tuning with a small amount of data and has actually been used in recent years for domain application of NE extraction (Hakala and Pyysalo, 2019; Li et al., 2020).

**FIGURE 4:**
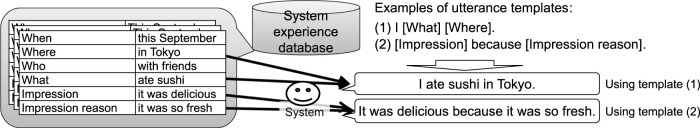
Utterance generation based on results of context understanding.

The dataset used to train the phrase recognizer is automatically generated from the system’s experience data and utterance templates. For example, given the utterance template “I [what] [where] [when],” the sentence “I ate sushi in Tokyo this September” can be generated by filling in each item from the system experience dataset as shown in Figure 4, and it is clear which part of the word sequence is *what* or *where*. Therefore, Begin, Inside, and Outside (BIO) tags for sequence labeling can be assigned automatically as {OB-WHAT I-WHAT O O O O} for the sentence “I ate sushi in Tokyo this September” if the *what* item is the extraction target. We made eight possible template patterns, for example, “I [what] [who]” and “I [what] [impression],” to establish a training dataset. The size of the established dataset is 213,204, and the details are written in Section 4.2.

### 3.4 Construction of a dialogue control module

This module has two functions. One is to control the dialogue flow and decide the next utterance template. The other is to find a system’s experience data which are similar to those of the user’s experience.

In order to maintain a reasonably natural conversation, the flow of the conversation was divided into small segments (first asking about *when, where, who, what*, then asking impression, and finally conveying empathy) and repeated it recursively by changing the contents (subtopic in conversation) related to the previous context, such as from famous spot to famous food under a *traveling* topic. Whether to ask a question at that time is simply determined by whether the target item of the frame is filled in.

The types of utterances are as follows. The simple empathy utterance and the follow-up question are used in pairs. When the *impression* slot is filled with the user’s experience data, the empathy utterance with evidence and the follow-up question are substituted.

#### 3.4.1 Simple empathy utterance

We prepared a list of simple utterances, such as “good” or “I see,” as in the conventional studies (Higashinaka et al., 2008; Kawahara, 2018), and a list of utterance templates for questions and empathy using experiences.

A simple empathy utterance is selected based on whether the sentiment of preceding user utterance is recognized as positive or negative. This sentiment recognizer is simply developed by a handcrafted rule, such as “I enjoyed xx” → *positive* or “I couldn’t go with xx” → *negative*. Specifically, we prepared lists of positive/negative words in advance to classify the sentiment. If a word was found in the preceding user utterance that matched the list, the utterance was assigned to that sentiment. The list of positive words included the words “happy” and “joyful,” and the list of negative words included the words “sad,” “disappointed,” and “could not go.” The vocabulary to be included in the list was chosen for each template of the system utterance, as it could vary depending on the question that immediately preceded the system utterance.

#### 3.4.2 Utterance to show the evidence for empathy

To show the evidence for empathy, we prepared utterance templates, such as “[impression] because [impression reason]” or “I [what], and [impression].” “The view from there was so beautiful because it has been hard to climb up here,” and “I climbed to the top of the mountain and the view from there was so beautiful” are examples of utterances made by filling the templates using the experience dataset.

#### 3.4.3 Follow-up question

We also prepared a list of follow-up questions, which includes not only such 5W1H+I questions as “Where did you go?” and “When did you go?” but also factual inquiries such as “Did you visit the Eiffel Tower?” and “Did you eat sushi?” For the former questions, the user’s 5W1H slot data can also be used as “Where did you go in [(user’s) where]?”, and for the latter questions, we can use the question template “Did you visit [where]?” or “Did you [what]?” to use the system’s experience data. Since only the names of places and foods are sometimes used for the template, we tagged them with [] as *ate* [*sushi*] so that these parts in brackets could be extracted later when creating the system’s experience dataset.

To find a system’s experience which is similar to the user’s experience, we use an original method to compute the similarity between the data of users and those of the system. The similarity is calculated by the sum of matched items, and it is highest when all items in the 5W1H+I are the same. Since it does not often happen that all items are the same, a similarity weight was assigned when matching is performed. For instance, we gave a higher priority to the contents of *what* and *where*. We also provided a constraint that at least either *what* or *where* must be extracted from the user’s experience and must be similar to the system’s experience.

Although we basically just count exact matches to determine whether an item matches, we also introduced a method of partial matching. In practical use, information about local amusement parks that are not included in the system’s experience data, or famous amusement parks, may be spoken, but their names may not be mentioned. Therefore, if there is a well-known amusement park within the region that appeared in the user’s conversation, that amusement park is selected. We explain elsewhere that the experience data of the system should be divided by region. If the user mentions an amusement park whose name is unknown to the system, the system selects the experience of visiting another amusement park.

Using a selected system experience similar to the user’s experience, the system can automatically generate an utterance by placing the experience data into an utterance template as shown in Figure 4.

### 3.5 Construction of an utterance generation module

This module receives system experience similar to the user’s experience and utterance template from the dialogue control module and automatically generates an utterance by placing the experience data into an utterance template, as shown in Figure 4. This module does not perform anything particularly difficult since it basically just completes the utterance with the experience data after receiving both utterance and experience data from the dialogue control module. If there are no replacement symbols in the template, this module simply outputs the inputted utterance as it is. For the generation of empathy utterance, there are several templates of patterns, which we describe in the following section.

By using our system design and system experience dataset, it is possible to construct a system using four sentence patterns with different ways of showing evidence when expressing empathy. The first two use experiences as one’s own experience (OwnExp) and hearsay experience (HearsayExp) to show evidence of the system’s experience; the other two use the impression reason of one’s own and hearsay experience (OwnExp+Reason, HearsayExp+Reason) to directly show evidence of the system’s feelings as follows.

**OwnExp** chooses a system experience that is similar to the user’s experience and mentions this experience and impression. For example, if the user says, “I saw Sagrada Familia,” the system chooses experience data and generates the following: “Oh, I went to Sagrada Familia in September, too. I really enjoyed it.”

**HearsayExp** is different from OwnExp from the viewpoint of how the experience is conveyed. Here, the system describes the experience as knowledge in the manner of hearsay experience. For example, “I learned that the place is enjoyable because I read some reviews on the internet describing it.”

**OwnExp+Reason** shows empathy based on the system’s own experience and impression using the same strategy as OwnExp, and it additionally mentions a reason for the impression. For example, when the same utterance “I saw Sagrada Familia” is given, the system says, “Oh, I also visited Sagrada Familia. I really enjoyed it because it is such a great building.”

**HearsayExp+Reason** also shows empathy based on the system’s hearsay experience and impression using the same strategy as HearsayExp, and it additionally mentions a reason for the impression. For example, when the same utterance “I saw Sagrada Familia” is given, the system says, “I’ve heard that the place is enjoyable because I read reviews on the internet describing it as a great building.”

By comparing the aforementioned four systems, it is possible to evaluate which empathetic utterances improve the user’s impression of “perceiving empathy.”

## 4 Experiment design

To evaluate our empathetic utterance design and the way to express empathy, we developed five types of chat systems: a baseline method and four extended methods using our system’s experience dataset: OwnExp, HearsayExp, OwnExp+Reason, and HearsayExp+Reason. The comparison methods are described in Section 4.1, the preparation of the system’s experience/knowledge dataset is provided in Section 4.2, and the experiment procedure to evaluate cognitive and affective empathy is described in Section 4.3)

### 4.1 Baselines and method settings

To evaluate the effects and key points of sharing experiences or impression reasons, we developed five types of chat systems and compared them. First, we evaluated the effects of sharing experiences by changing the types of experiences (one’s own and hearsay), and then we compared the effects of explaining why the feeling surfaced using an impression reason. The condition is listed in Table 1.

**TABLE 1:** Comparison of system conditions for evaluation of sharing experiences and mentioning impression reason when sharing experiences.

Method	General fact	Own experience	Hearsay experience + Impression reason	
Baseline	✓			
OwnExp		✓		
HearsayExp			✓	
OwnExp+Reason		✓		✓
HearsayExp+Reason			✓	✓

The **baseline** method simply empathizes with the user through utterance repetition, as in conventional dialogue systems (Higashinaka et al., 2008; Kawahara, 2018). For example, if the utterance “I saw Sagrada Familia” is given, the system partially repeats the user’s utterance as “Oh, Sagrada Familia!” and adds a simple empathy utterance: “That’s good!” or “I see.” In addition, such facts or opinions as “Sagrada Familia is famous in Spain” are added to lessen the impact of the difference in the number of the system’s utterances. The completed utterance becomes “Oh, Sagrada Familia! That’s good! Sagrada Familia is really famous in Spain.” We prepared five types of simple empathy utterances, one of which was selected based on whether the preceding user utterance had a positive or negative sentiment. The sentiment was classified using the positive/negative words list described in Section 3.3. We used the same positive/negative word list in all conditions, including the baseline.

Since the effects of simple empathy utterances have already been shown in previous research (Higashinaka et al., 2008), we used the strategy of including simple empathy utterances as the baseline for this evaluation. It is important to note that the performance of the baseline system actually constructed shows response performance similar to that of recent neural-based dialogues (Section 6.1).

The four methods other than the baseline are OwnExp, HearsayExp, OwnExp+Reason, and HearsayExp+Reason described previously. For HearsayExp, although we can generate such phrases as “I heard the place is enjoyable from my friend who told me that she enjoyed Sagrada Familia” using our system’s experience, we do not use such phrases in this study because the expression suggests that this system has a relationship with others (humans). It has been reported that people relate to a robot as more human-like when they know that the robot has a relationship with others (Cordar et al., 2014). Therefore, we decided not to use these phrases to isolate this effect.

All of the developed dialogues were conducted based on the same dialogue flow, and the empathy utterances are different in each method. The dialogue contents change based on the user’s utterances. We compared the aforementioned text chat-based systems to evaluate the effects of sharing experiences depending on the expression types.

It is important to note that we used simple empathy utterances as a baseline instead of not using empathy utterances because the effectiveness of simple empathy utterances has been reported in the conventional research (Higashinaka et al., 2008). In addition, the interest in our work is the difference between methods of expressing empathy.

### 4.2 Preparation of a system experience dataset and user experience recognizer

To fit the utterance template described in Section 3, we developed the system’s experience dataset. We chose the *travel* topic as our corpus domain because it evokes ordinary conversation in human–human conversation (Arimoto et al., 2019) and includes many experiences. Our system’s experience dataset was created by hired workers who were given the following instructions: “Fill out the [when], [where], … items based on your experience or imagination. These items will be used as the contents of such utterance templates as ‘I visited [where] before.” We provided the workers with three kinds of utterance templates as examples. Since we chose *travel* as our corpus domain, creating conversations about famous tourist spots or famous foods is simple for each area by referring to guidebooks. The individuals who made the experience corpus mined their own memories of spots and filled in the 5W1H+I items and their reasons. If they had never visited a particular tourist spot, they filled in the items by imagining information based on the guidebooks or the web. It is important to note that although the workers knew that these data would be used to make the system’s utterances, they were asked to imagine and describe what a person (i.e., the worker himself/herself) would be able to experience, regardless of whether the system could or could not be implemented. Furthermore, since it was difficult to create a single template that could be universally used for all elements in *what*, we divided the elements of *what* into two categories and created a template for each category. Although we had initially divided the elements of *what* into the five categories, “see,” “learn,” “eat,” “experience,” and “buy,” which are commonly used in travel guides, we eventually settled on the two categories “eat” (food-related) and “other” because the template for natural utterances differs in them. We collected all data in Japanese for this experiment.

To allow the system to easily find experiences that resemble the user’s experiences, all data in the corpus include information on the name of a region, such as the name of a prefecture or a city, town, or village. Figure 2 shows an example of the experience/knowledge data in the dataset. Each data source in the experience corpus contains all of the area information, 5W1H+I items, and their reasons. We collected 2,652 data as shown in Table 2 for our experience corpus.

**TABLE 2:** One of the examples in the prepared experience dataset.

Item	Content
Area (prefecture)	Tokyo
Area (city)	Akihabara
When	In this summer vacation
Where	Electronics shop
Who	With friends
What	Bought electronics parts
Impression	It was exciting
Impression reason	There were various types of parts

For the 5W1H+I recognizer, we automatically generated a training dataset by using the collected experience corpus with sentence templates. We created eight possible template patterns as sentences such as “I [what] [who]” and “I [what] [impression]” and then filled each item into the sentence templates by changing the experience data in the experience corpus. The BIO tags were given at the same time, as shown in Section 3.3. The generated training dataset has 213,204 sentences with BIO tags. We adopted BERT for sequence labeling and a pretrained model trained with Japanese Wikipedia (Kikuta, 2019). We then fine-tuned the pretrained BERT using our dataset of 213,204 items for sequence labeling.

In preliminary validation testing, the detection accuracy for 5W1H+I items using the developed test dataset (size of dataset: 13,893) was 72%. Using this recognizer, the system can extract words or phrases for the 5W1H+I. We also confirmed that new types of phrases could be extracted as targets: *the park near Kyoto Station* is extracted as a location, even without a formal proper name, and *ate sushi* is extracted as a *what* item. The results extracted by the conventional named entity recognizer and those by our proposed phrase recognizer are shown in Table 3. With the recognizer, our developed systems understood the context by filling in the 5W1H+I frames through conversations.

**TABLE 3:** Comparison between location phrases extracted by the conventional method and by the proposed method.

User utterance (italic: location phrase)	NE extractor	Proposed phrase extractor
I went to *Italy* for summer vacation	Italy	Italy
I saw a rainbow at *the park near Paris Station*	Paris Station	The park near Paris Station
I often go to *electrical shops*	N/A	Electrical shops

When *the park near Kyoto station* is recognized as *where* element as a user’s experience, the system cannot find the exact matched location in the system’s experience/knowledge dataset. Therefore, the system relaxes the search condition and searches for *where* elements in the system experience data with partial matches instead of exact matches. Finally, the system chooses an experience about a park without a named entity and conveys empathy using the experience at the park in this other location as an experience that resembles the user’s experience.

### 4.3 Experiment procedure

We evaluated our developed systems through user evaluations. We hired crowd workers as examinees who can speak Japanese at the everyday conversation level to evaluate the systems. They chatted with the five systems described in the previous section using Telegram^2^, which is a text-chat messaging tool, and subjectively evaluated each system with questionnaires.

The following is the experimental procedure for each examinee: 1) the examinee answered questions about user characteristics. We asked about gender, familiarity with chat systems, and expectations of chat systems, but we did not use the results in this study. 2) The examinee chatted with the first system by text chat. 3) The examinee rates the system’s performance by answering the questionnaire described in Table 4. 4) Procedures (2) and (3) were repeated five times for each chat system. Each chat consisted of 30 utterances, 15 from the system and 15 from the users. The system said thank you and ended the conversation after the user input 15 utterances to the system. The examinees chatted with all five systems. To avoid any order effect, the order of the conversation systems was randomly determined. Normally, an equal number of participants can be assigned to any order by randomly assigning them in advance. However, in this experiment, a random assignment was chosen because participants were allowed to leave in the middle of the experiment and system constraints made it impossible to assign the assignments of those who left in the middle to new participants.

**TABLE 4:** Questions asked to users who have had direct text chats with the dialogue system to evaluate the system. Q2 measures the impression of the system’s cognitive empathy and Q3 measures that of one’s affective empathy. These questions were answered on a 7-point Likert scale (1–I do not agree at all, · · ·, 7–I totally agree). Q1–Q4 are used for the main analysis, but Q5 is listed here because it is used in the interpretation of the results.

Qid	Notation	Question (To what extent do you agree with the following statements?)
Q1	TalkOwnExp	The system chatted based on its own experience
Q2	Understanding	The system understood what you were chatting about
Q3	Empathizing	The system empathized with you
Q4	Satisfaction	You satisfied with the chat conversation with the system
Q5	Reliability	The system was reliable

The examinees were instructed as follows: *Chat with five different AI robots in text chat and evaluate each system in turn. Start from “Hello” input. Use multiple utterances in a single input to take your turn. Please chat with the AI robots freely and as naturally as possible, as you would speak with humans.* We then showed a list of tourist spots or food in each area with which the system was familiar in case the user does not remember any memories about his/her travel. Although our examinees were given the list, they were allowed to mention places, food, or other information outside of the list because we prioritized having the examinees chat freely and naturally without any feelings of constraint.

We prepared the four questions listed in Table 4, which were chosen for the following reasons: Q1 (the system chatted based on its own experience) is asked to verify whether the proposed system is designed as intended. Q2 (impression of understanding), which measures the cognitive empathy, evaluates the impressions of the system’s ability, and Q3 (impression of empathizing), which directly measures the affective empathy, evaluates the scores that directly indicate the specific effects related to the purpose of our study. Q4 (satisfaction) is derived from questionnaires that are often used to evaluate chat dialogue systems (Finch and Choi, 2020). In addition to the aforementioned questions, other questions were asked about users’ impressions of the dialogues and personality traits, but these are not listed because they are not used in this study. In the preliminary examination, we also asked “You enjoyed the chat conversation with the system” and “You want to chat with the system again” and found that they were highly correlated to Q4. Therefore, we used Q4 to evaluate the secondary effects of showing about the system’s experiences. Each was evaluated using a 7-level Likert scale (Allen and Seaman, 2007), where score 1 is lowest and score 7 is highest. To evaluate the significant differences among the scores, we calculated the *p*-value using Welch’s *t*-test.

We hired 58 examinees (29 men and 29 women) ranging in age from their 20s to 50s and had them evaluate the five systems. We conducted a preliminary experiment using another chatting task and selected 58 examinees who worked diligently without cutting corners for the purpose of screening. They repeated chatting with a system and answered the questionnaire five times. Since dialogue histories were submitted with each questionnaire, we collected 290 dialogue histories and the answers to the questionnaires.

Since we did not set any constraints, as a way to prioritize having examinees speak freely with the system, it is possible that some places, foods, or other information mentioned by the examinees fell outside of the system’s experience. In this situation, the system might confuse the mentioned place with a similar, but different, place that it knows. Hence, it was difficult to produce the implied empathy utterances because non-similar experiences were shared. This can also lead to dialogue breakdowns where the system and the examinee mention different places and create a situation in which the user feels that “the system does not understand me.”

Therefore, we analyzed the ratio of dialogues having dialogue breakdowns after the experiment. We hired an annotator to judge whether the conversation had dialogue breakdowns. The annotator was trained in advance to accurately annotate dialogue breakdown labels defined in the research (Higashinaka et al., 2015), and she then judged whether the conversation had dialogue breakdowns by checking the dialogue logs. The dialogue breakdowns include a repetition of the previous utterance, an utterance inconsistent with the previous utterance, an abrupt utterance, an utterance that ignores the users’ utterance including not answering to questions, and an utterance that contains typographical or grammatical errors. Thus, minor breakdowns such as grammatical errors are also included. The main critical dialogue breakdown was that the system told an experience about a different tourist spot than the one the user had mentioned. The reason was that the system could not find an experience similar to the one mentioned by the user.

Therefore, the system misunderstood what the user mentioned and ended up that the conversation did not mesh. The ratios of dialogues with dialogue breakdowns in each method are as follows: Baseline: 0.62; OwnExp: 0.65; HearsayExp: 0.57; OwnExp+Reason: 0.64; and HearsayExp+Reason: 0.62. The difference between the aforementioned ratios ranges from 0.03 to 0.08 points. Since the maximum difference is 5 (= 58 dialogues ×0.08), it would say that it is not a big deal.

## 5 Evaluation results

### 5.1 Effects of showing experience to convey empathy

**FIGURE 5:**
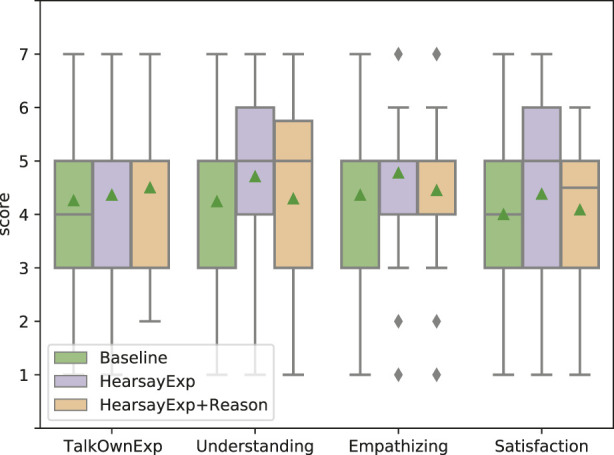
Effectiveness of showing experience compared with baseline. Each bar colored green, purple, and orange shows the results of baseline system, OwnExp system, and HearsayExp system, respectively. TalkOwnExp, Understanding, Empathizing, and Satisfaction along the *x*-axis represent the question items (corresponding to the notation in Table 4). Triangles represent mean values in all, and diamonds represent the outlier.

Figure 5 shows the results of an evaluation of how users’ impressions change depending on whether the experience conveyed as the basis for empathy is conveyed as one’s own or as hearsay. Though there is no significant difference in the three systems for TalkOwnExp and Satisfaction, there are significant differences between OwnExp and HearsayExp in the results for Understanding (*p* = 0.030 *<* 0.05) and between OwnExp and HearsayExp for Empathizing (*p* = 0.034 *<* 0.05). These results suggest that the hearsay experience was deemed reliable as the system’s utterance because it was close to what the dialogue system could actually act on. In fact, many of the dialogue systems on the market are connected to the internet, and the systems themselves can obtain information via the Web; therefore, hearsay experience seems to be the system’s natural act. In addition to this, there was a strong correlation between the scores of empathizing and reliability in terms of the actual user impression (*R* ^2^ = 0.632). Therefore, it can be said that the hearsay experience that the system provides as the basis for empathy contributes to improving the users’ impression of reliability of the system and also improving the users’ impression of system’s skill of cognitive empathy (understanding) and affective empathy (empathizing). As a further argument, it may seem odd that despite the improvement in empathy, the satisfaction score did not improve. One possible reason for the lack of improvement in satisfaction is that the large variance in satisfaction scores suggests that users’ motivations regarding their expectations of the system may have varied from person to person.

### 5.2 Effects of showing impression reason

**FIGURE 6:**
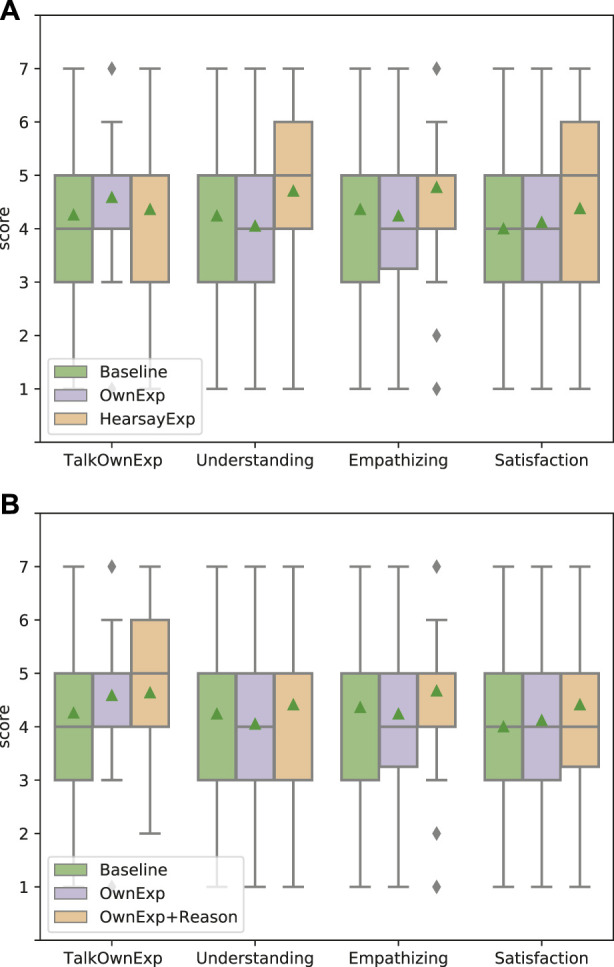
Effectiveness of showing the impression reason of experience compared with baseline and only showing experience. Each bar shows the results of baseline, showing own/hearsay experience without reason (OwnExp/HearsayExp), and showing own/hearsay experience with reason (OwnExp+Reason/HearsayExp+Reason). Triangles represent mean values in all, and diamonds represent the outlier. In the case of OwnExp, the scores of “chat based on system’s own experience” (TalkOwnExp) and Empathizing are higher when the impression reason is included, and the score of Empathizing is higher. On the other hand, in the case of HearsayExp, the score of Empathizing is higher regardless of whether the impression reason is included. **(A)** Apply to talking own experience system. **(B)** Apply to talking hearsay experience system.

We also evaluated the effectiveness of talking about the experience impression reasons (Figure 6). To examine whether the effect differs depending on the method of conveying experience, we evaluated two patterns: adding impression reason while showing own experience (OwnExp and OwnExp+Reason) (Figure 6A) and adding impression reason while showing hearsay experience (HearsayExp and HearsayExp+Reason) (Figure 6B). The results of OwnExp and OwnExp+Reason show that the mean values in the impression of “The system talks its own experience (TalkOwnExp)” are 4.579 and 4.635, respectively, and OwnExp+Reason is highest in all, but the difference between OwnExp and OwnExp+Reason is not significant (*p* = 0.831 *>* 0.1). Therefore, it can be said that the inclusion or exclusion of impression reason does not affect whether the user feels that the system is talking about its own experience. Then, they also show that the mean values in the impression of “The system empathizes with me (Empathizing)” are 4.245 and 4.667, respectively, and OwnExp+Reason is highest in all. Although the difference between OwnExp and OwnExp+Reason for Empathizing was not obviously significant, it tended to be significant (*p* = 0.096 *<* 0.1). This result indicates that including the impression reason to convey system’s own experience improves somewhat the user’s impression of empathizing. This suggests that the explanation of their own experience and their impression reasons may have led humans to think that “the system may actually have feelings.” Although we do not know how human thinking led to improve their impressions, we show the possibility that adding the impression reason with their own experience is effective in improving the impression of empathizing when the system expresses empathy based on their own experiences. Although there was no significant difference between Understanding and Satisfaction in the case of OwnExp regardless of the reason, a similar trend was observed for Understanding and Empathizing in terms of the mean values. This tendency was also observed in the case of HearsayExp.

The results of HearsayExp and HearsayExp+Reason show that there are no significant differences among all scores. In terms of score comparisons, overall, the scores of talking hearsay experience (HearsayExp) are higher than those of including the impression reasons (HearsayExp+Reason). These results suggest two conclusions: first, the impression can be sufficiently improved by using hearsay experiences as the basis, even without the corresponding impression reasons. Second, it seemed unnatural for hearsay experiences to include impression reasons, even though they were not experienced by the system itself. Although not a rigorous analysis, the scores for Understanding and Empathizing were inversely reduced by adding the Impression reason, suggesting that the inclusion of the impression reason may have been unnatural with the hearsay experience.

To summarize the aforementioned results, showing hearsay experiences as a rationale to convey empathy improves the impression of “the system understands/empathizes with me.”

**FIGURE 7:**
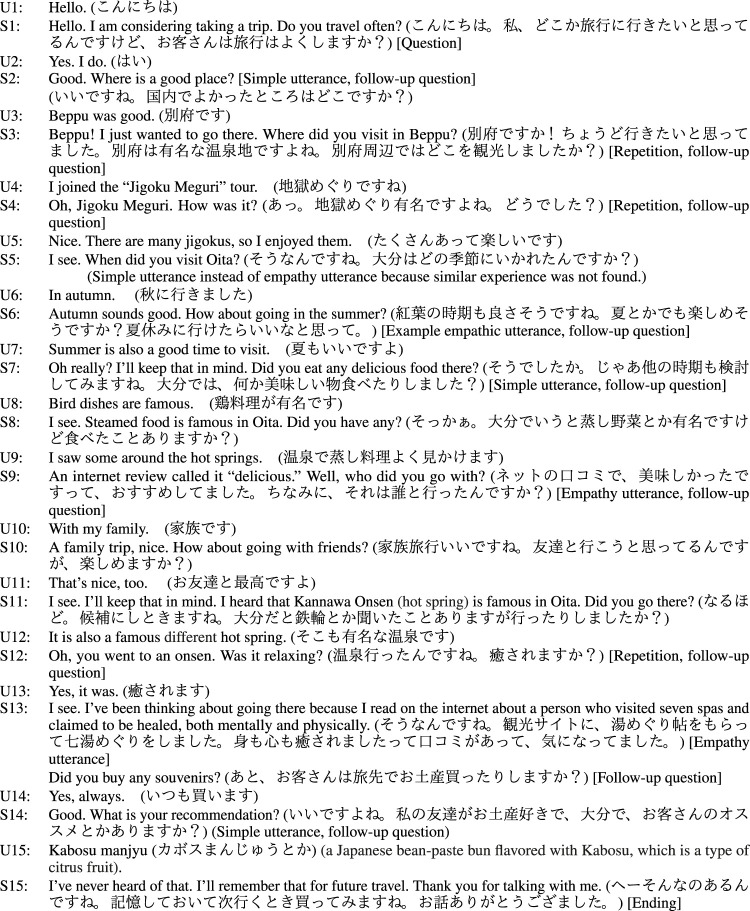
Dialogue history with user (U) and system (S) with HearsayExp. Jigoku Meguri, referred to in U4 and S4, is a famous hot spring in Oita prefecture. S5 is related to U3 because Beppu is located in Oita prefecture.

Finally, we discuss the evaluation results by showing the example of dialogue history shown in Figure 7, which is an actual dialogue history translated in English with a user and a system with HearsayExp. The maximum number of empathic utterances that occur in the dialogue is 3. However, this number changes if the system cannot find experiences that resemble the user’s. When the utterance is indicated in bold letters, the system cannot find a suitable experience and only provides a simple utterance. The impression of empathy can be further improved if the system finds a suitable experience every time. Therefore, automatically increasing the experience data in the experience corpus through real human–agent conversation or extracted from human–human conversation is our next challenging work. This work is challenging because of the difficulty in determining whether the information obtained from the user’s utterance is truly correct.

## 6 Verification and analysis

In interpreting the evaluation results, we further evaluated the performance of the baseline system by comparing state-of-the-art conventional dialogue systems and the effects of using rationale to convey empathy using another analysis method based on multiple regression analysis. We recruited 20 annotators for this evaluation.

### 6.1 Performance of a baseline system

In interpreting the evaluation results, it is desirable that the performance of the baseline system is not too far from the performance of recent dialogue systems. Therefore, we asked the annotator to evaluate the performance of the baseline system and the system including the recent neural base. Specifically, we compared our baseline dialogue system to the first- and second-place systems, HBY (Sugiyama et al., 2020) and ILY (Fujihara et al., 2020), respectively, in a competition to evaluate the performance of Japanese dialogue systems (Higashinaka et al., 2020).

Since the system itself is not publicly available, we used the dialogue histories with users in the preliminary and final rounds of the competition, which are publicly available, for evaluation. The annotators were asked to review the dialogue histories and give answers on the level of system satisfaction, understanding, and empathizing using a 5-point Likert scale for the same questions used when they evaluated our system. Moreover, we asked them to make this evaluation assuming they were the users who had conducted the dialogues. Each dialogue history was evaluated by five annotators. The reason for using a 5-point Likert scale is that it is considered difficult to make an evaluation at a fine level of detail in a situation where the user is not interacting directly with the dialogue systems.

**FIGURE 8:**
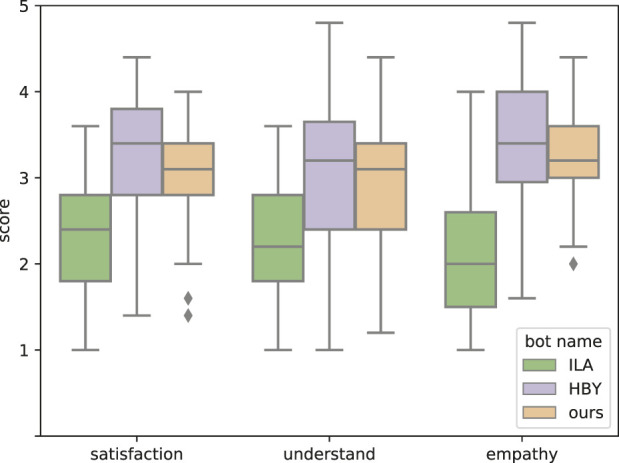
Performance of our baseline system. The score corresponds to the ratings on the 5-Likert scale for “Willing to talk again.”

Figure 8 shows the comparison results of our baseline system and other conventional neural-based Japanese dialogue systems, which were the state-of-the-art systems at the time. Our baseline performance is close to that of the first-place system in any of these items, while HBY was the first-place system in the competition and ILA was the second-place system. Therefore, it is assumed that we were able to demonstrate the effectiveness of empathic utterance design in a system with a certain degree of natural response.

### 6.2 Effects of using rationale to convey empathy

To examine the kinds of utterances that had an effect on improving the user’s satisfaction, in terms of the impressions of understanding and empathy, we annotated each utterance of the dialogues used in the evaluation. In the annotation, all utterances were assigned regardless of whether they corresponded to the following eight labels, and then we analyze how much the number of utterances that corresponded to each label contributed to the impression of the dialogue by multiple regression analysis.

The labels to be annotated are as follows. (L1) The utterance contains a rationale. (L2) If the utterance contains a rationale, the rationale is the robot’s own. (L3) The utterance includes the system’s own experiences. (L4) The utterance includes Other’s experiences. (L5) If the utterance includes experiences, the system’s experience is similar to the user’s experience. (L6) The utterance includes the system’s feelings. (L7) If the utterance includes the user’s feelings, the feelings are similar to the user’s feelings. (L8) The utterance shows empathy for the user. For each item, a label was assigned as True or False.

The 20 hired annotators were each randomly assigned dialogue history files in such a way that five annotators could work on each dialogue history. The annotators assigned labels to all system utterances in the dialogue. Basically, the instructions were as described previously. For L4, we defined the experiences of people other than the system (e.g., experiences seen or heard by the system) as the experiences of others and instructed that hearsay and other experiences should be True. L2, L5, and L7 were annotated only if L1, L3, L4, or L6 were True. In addition to this, we asked the annotators to rate their impressions of the dialogue, as if they were the users of the dialogue. The question items were common “To what extent do you agree with the following statements?” with “the user satisfied with this dialogue” (Satisfaction), “the system understands the user’s utterance accurately?” (Understanding), and “the system empathizes with the user?” (Empathizing), as well as items rated by actual interlocutors. We asked them to answer the questions as if they were the users of this dialogue. Although it is possible to analyze the relationship between the evaluation scores of actual interlocutors with the labels annotated on each utterance, the analysis method does not allow us to identify direct relationships between L1∼L8 and impression scores. Since there is a direct relationship between the perception of an utterance and the impression of the dialogue, we consider it necessary to evaluate the impression of the dialogue by the same person who made the annotation. In addition, since only one rating score can be assigned by an interlocutor, but multiple people can rate the same dialogue in annotation, the average of their scores can be used to ensure the validity of the score. Therefore, we analyzed the relationship between the impression scores of the dialogues evaluated by the annotators and the labels assigned by the annotators. The data to be annotated are all 393 dialogues, including baseline and comparison methods. The total number of annotated dialogues is 1,965.

We used multiple regression analysis to examine the contribution of the number of utterances in which each label was True to the impression of the dialogue, i.e., satisfaction, understanding, and empathizing. Response variables are the scores of satisfaction, understanding, and empathizing. All annotated labels were used as explanatory variables. To investigate the effectiveness of including evidence and system experience in the empathetic utterances, we added the labels L1′–L7′, which represent the AND conditions for L8, and each L1–L7, to the explanatory variables. Thus, the total number of explanatory variables was 15. Since the sample consists of annotated dialogues 1,965, the number of explanatory variables is considered reasonable. For the model of multiple regression analysis, the best-fitting model was selected using Akaike’s information criterion (AIC) (Akaike, 1987) for each response variable.

Table 5 shows the results of multiple regression analysis. The table shows only those cases in which there is a significant difference or trend or correlation to some extent. From the results, it was observed that utterances showing empathy improved impressions of satisfaction, understanding, and empathizing for the dialogue. In addition, incorporating hearsay experiences in the utterances contributed more to improving impressions than using the system’s own experiences in the utterances. In the hearsay experience, the experiences that the system utterances are within the range where the system can actually act may have contributed to the improvement in the impression by increasing the credibility of the system’s utterances. It was also suggested that the presentation of a simple rationale makes a substantial contribution to satisfaction, and that the presentation of a rationale when empathizing improves the impression of empathy as “the system understood me,” which represents cognitive empathy. Furthermore, the results of L6 suggest that including the system’s own feelings when empathizing can improve the overall impressions for conversation.

**TABLE 5:** Results of multiple regression analysis. *β* represents a standard partial regression coefficient, and *r* represents a correlation coefficient. Only explanatory variables for which significant differences were found in any of the items are listed. Bolded figures indicate those with significant differences in *β* and a correlation coefficient *r* of 0.3 or more.

Explanatory variables	Satisfaction		Understand		Empathy	
	*Β*	*r*	*β*	*r*	*β*	*r*
(L2) System’s own rationale	**0.077** ^*∗∗*^	**0.337** ^*∗∗∗*^	0.086^*∗∗∗*^	0.291^*∗∗∗*^	0.045	0.325^*∗∗∗*^
(L3) System’s own experience	0.056	0.006	0.010	−0.045	0.070^*∗*^	0.014
(L4) Hearsay experience	**0.090** ^*∗∗*^	**0.321** ^*∗∗∗*^	**0.091** ^*∗∗*^	**0.315** ^*∗∗∗*^	**0.140** ^*∗∗∗*^	**0.353** ^*∗∗∗*^
(L5) Similar to user experience	−0.332^*∗∗∗*^	0.0150	−0.367^*∗∗∗*^	−0.008	−0.252^*∗∗*^	0.045
(L8) Show empathy	**0.117** ^*∗∗∗*^	**0.569** ^*∗∗∗*^	**0.076** ^*∗∗*^	**0.510** ^*∗∗∗*^	**0.132** ^*∗∗∗*^	**0.616** ^*∗∗∗*^
(L1′) Empathy with rationale	0.284	0.414^*∗∗∗*^	**0.447** ^*∗∗*^	**0.365** ^*∗∗∗*^	0.098	0.425^*∗∗∗*^
(L2′) Empathy with own rationale	−0.386	0.348^*∗∗∗*^	−0.622^*∗∗*^	0.261^*∗∗∗*^	−0.248	0.345^*∗∗∗*^
(L6′) Empathy with own feeling	**0.190** ^*∗∗∗*^	**0.464** ^*∗∗∗*^	**0.235** ^*∗∗∗*^	**0.437** ^*∗∗∗*^	**0.224** ^*∗∗∗*^	**0.494** ^*∗∗∗*^
R-square	0.415		0.384		0.508	
Adjusted R-square	0.408		0.373		0.499	

## 7 Discussion

The fact that the average scores in Figures 5, 6 are close to 4–5 out of 7 ratings and that the difference is about 1 needs to be discussed. A difference of 1 out of 7 ratings does not seem very large, but given the context of the dialogue, even this small difference is important. Unlike counseling situations, where much of the usual research on empathy has been conducted, the present experimental situation deals with an everyday conversational situation in which empathy is not necessarily required. This situation can be seen as one where empathy is not given much attention and where the ability to empathize is taken for granted. This will also be discussed in the following sentences. In addition, the reason why the values are in the middle of the range 4–5 may be due to the fact that it is considered ‘natural’ among people to be able to empathize with others. In this experiment, we assumed that the baseline system was also empathetic in order to prevent unfair comparisons. Thus, while all systems received somewhat high overall scores, it is clear that systems that only ask questions of the user without empathetic utterances were given 2 or 1 points.

This can be considered to indicate the severity of the user’s evaluation of empathy in daily conversation.

**FIGURE 9:**
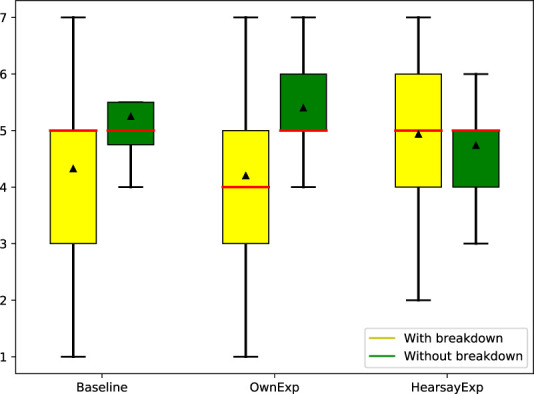
Empathizing score of Baseline, OwnExp and HearsayExp with/without dialogue breakdowns.

Since the main focus of this study was to have users’ experience an actual chat with the system and evaluate their impressions of the system at that time, some dialogue breakdowns or errors could have occurred during the conversation if the system misinterpreted the users’ utterances. However, as shown in Section 5, the actual dialogue history did not show any significant differences between the compared systems in terms of the presence or absence of dialogue errors during the conversation, so these effects could be ignored in the analysis. To further explore the detailed conditions which are effective using system’s own and hearsay experience, we additionally analyzed the difference in ratings of the impression of empathizing in two patterns: Dialogue including at least one breakdown (with dialogue) and Dialogue without such a breakdown (without dialogue breakdown) (Figure 9). The results show that HearsayExp is highest in dialogues with breakdowns and OwnExp is highest in dialogues without breakdowns. This means that the results confirm what has been said in studies of human conversation (Batson et al., 1996), namely, that sharing experience (OwnExp) is effective because human conversation does not include dialogue breakdowns. In addition, it is suggested that HearsayExp is effective under conditions that may include dialogue breakdowns, such as in a dialogue system. It is important to note that dialogue breakdowns here include not only those that are understood incorrectly or ignored but also simple grammatical errors and typos so that people can understand how they made a mistake. Therefore, in order to verify the effect of a breakdown, it is necessary to have dialogue data in which the breakdowns included in the dialogue are of a single type as much as possible, which is not easy to evaluate. Such an evaluation would be possible if a large amount of dialogue data and annotation could be performed.

The domain of the system used in this study was *travel* and was limited to destinations in Japan that fit into a realistic amount of the experience dataset for manual creation. However, the phrase extractor presented in this paper has the potential to easily and automatically extend the system’s experience database. The phrase extractor is able to extract the words or phrases corresponding to 5W1H from a sentence. Thus, as long as we can detect a segment of sentences about an experience from a blog or an article on the Internet, we can automatically fill in the 5W1H experience data, which can then be used as the system experience database. Similarly, although the system can use the 5W1H slots extracted from conversation with users as the system’s real hearsay experience data, it is a challenging because the extracted slot information is not always correct.

In this study, we used a rule-based system to examine how users’ impressions change depending on how the system conveys empathy. However, the results of this study may also be useful for neural network-based dialogue systems. For example, since our method can automatically generate the system’s utterances given the system’s experience database and utterance templates, the automatically generated utterances could be used as training data for a neural-based dialogue system. Furthermore, since some methods for converting speech styles have recently been proposed (Fu et al., 2018), it may be useful to convert the utterance generated by the neural-based dialogue system into an empathetic utterance. We expect that these results will be verified in various dialogue systems in the future.

On the other hand, evaluating the effectiveness using neural-based methods may not be easy. In this experiment, since the used rule-based method has the same types of dialogue breakdowns that can occur even after repeating conversations with users and there was no significant difference in the number of breakdowns between methods, we can evaluate the effectiveness of the ways to convey empathy without considering breakdowns. However, in neural-based methods, the types and number of possible breakdowns may vary from one dialogue to another, which may affect the effectiveness of the proposed method. In this case, it may be necessary to evaluate a sufficient number of dialogues and users to neglect the variations in the breakdown. It would also be useful to analyze in detail how the type and number of breakdowns affect the user’s impression. In this case, since it is difficult to control the system according to a certain number of breakdowns, the effectiveness of the utterance could be evaluated using the dialogue logs to evaluate the user’s impressions and annotate breakdowns after a user has conversed with the system.

## 8 Conclusion

To explore the effective empathetic utterance design to improve the user’s impression of receiving empathy, we focused on *making users grasp the robot or agent as an Other who can experience the entity* and proposed a dialogue system and system utterance design to directly share it in text base conversation. Our proposed system shows empathy based on a system’s own and hearsay experiences that resemble a user’s experiences as an evidence of empathy. The exhaustive evaluations showed that a human impression of understanding improves when the system shows empathy using the system’s hearsay experience. They also showed that when showing the system’s experience, the system does not need to go into the impression reasons and mention them.

The design of this utterance is template-based and very simple, making it applicable to rule bases for commercial use. We only adopt rule-based methods to verify the effect of utterance design in this study. In future work, we will automatically create the system’s experience data from human–robot conversation or from general articles, and then we will examine whether the same effect can be obtained by fine-tuning a neural-based generative model using the data created by automatically filling the experience data to the utterance templates.

## Footnotes

1 We used the BERT model pretrained using Japanese Wikipedia (Kikuta, 2019).
